# High-intensity focused ultrasound for the treatment of allergic rhinitis using nasal endoscopy

**DOI:** 10.3892/etm.2012.798

**Published:** 2012-11-05

**Authors:** LIANG-JUN CHENG, BING LIU, BO NING, HAO MING, CHI WANG, LI-XIA WAN

**Affiliations:** 1 Otolaryngology Head and Neck Surgery Department of Xuzhou Central Hospital, Jiangsu;; 2 Otolaryngology Head and Neck Surgery Department of Xuzhou Medical Hospital, Jiangsu;; 3 Daizhuang Hospital of Jining, Jining 272029, Shandong, P.R. China

**Keywords:** allergic rhinitis, focused ultrasound

## Abstract

The present study aimed to observe the therapeutic effect of high-intensity focused ultrasound for the treatment of allergic rhinitis (AR) using nasal endoscopy. A total of 72 patients with perennial AR received treatment using the CZB ultrasonic therapeutic instrument with nasal endoscopy. A scoring method was adopted for evaluation of effectiveness according to the AR therapeutic principles and recommendations described in Allergic Rhinitis and its Impact on Asthma (ARIA) in 2001. The patients were followed up between 2 and 6 months after treatment. The excellence rate was 34.7% (25/72), the effective rate was 62.5% (45/72) and the ineffective rate was 2.8% (2/72). The total effective rate reached 97.2% high (70/72). Endoscopic high-intensity focused ultrasound for the treatment of AR is a non-invasive method and has the advantages of simple manipulation, a short course, high safety and a clear short-term effect.

## Introduction

High-intensity focused ultrasound was developed originally by Lynn *et al*(1) in 1942. It is a non-invasive and safe therapy with great potential. At present, it has been applied in an increasing number of areas, including oncology, urology, ophthalmology, brain science and otorhinolaryngology (2–6).

Allergic rhinitis (AR) is an allergic disorder occurring at the nasal mucosa and has the manifestations of rhinocnesmus, sneezing, excessive nasal discharge and nasal mucosal swelling. It is a common disease in otolaryngology. In addition, it bears close correlations with bronchial asthma, nasal sinusitis and conjunctivitis, and is one of the risk factors for asthma (7,8). In recent years, the incidence of AR is increasing, which markedly influences the quality of life of patients (9). However, its pathogenesis remains unclear. AR is an obstinate disease to which simple drug treatment does not provide satisfactory relief.

Between January and June 2009, 72 AR patients underwent endoscopic high-intensity focused ultrasound in the Otolaryngology Head and Neck Surgery Department of Xuzhou Central Hospital and Affiliated Hospital of Jining Medical College. The patients were followed up between 2 and 6 months after treatment. A clear therapeutic effect was observed.

## Patients and methods

### Clinical data

All patients (n=72) involved in the current study met the revised diagnostic criteria of perennial AR described in Allergic Rhinitis and its Impact on Asthma (ARIA) in 2001 (10,11). Among the patients, 45 were male and 27 were female, with an average age of 35.6 years (range, 18 to 65). Patient AR history ranged from 1 to 25 years with an average of 8.64. The majority of patients had received long-term drug treatment but without satisfactory therapeutic effectiveness. The patients had not received nasal surgery.

### Methods

The patient was placed in a supine position. Anesthesia was performed on the rhinal surface with cotton pellets which were dipped in 2% tetracaine and 1% adrenaline. All sites requiring treatment were anesthetized sufficiently, particularly the posterior upper border of the inferior turbinate, the site where branches of the sphenopalatine nerve extend into the inferior turbinate and the site where the anterior ethmoidal nerve extends into the nasal cavity. The anesthesia was performed 3 times for 10 min in total. CZB ultrasonic AR therapeutic instrument (Chongqing Hifu Co., Ltd., Chongqing, Sichuan, China) was applied and the power parameter was set to Gear III. The ultrasound emitter window was placed against the bilateral inferior turbinates and the corresponding nasal septa. The entire range of the inferior turbinate (including the free, medial and upper borders) and the anterosuperior part of the nasal septum were scanned from top to bottom and then from back to front in a Z-formed tour. The speed was set at 3 mm/sec and each site was treated twice. The total treatment time was 500–600 sec. No bleeding occurred after treatment. The nasal cavity was flushed with saline immediately. Prednisone acetate tablets were administered orally for 3 days in order to relieve nasal mucosal swelling, pain and inflammatory reactions. Patients received follow-up and nasal discharge removal 3 days after treatment.

### Evaluation of effectiveness

Therapeutic effectiveness was evaluated according to the symptom-based scores, the AR therapeutic principles and recommendations and signs and symptoms. The indices of therapeutic effectiveness were calculated based on the following formula: Effect index = (the total score before treatment - the total score after treatment)/the total score before treatment × 100 (≥66 was considered to be excellent, ≥25 - <66 was considered effective and <25 ineffective).

## Results

The average treatment time was 592 sec and the average treatment power was 1,947 W. The patients suffered from swelling at the inferior turbinate and nasal septum with obvious nasal congestion and yellow jelly-like discharge following treatment, particularly on days 1–3. One week later, the symptoms of rhinocnesmus and nasal congestion were markedly relieved, sneezing and running of clear nasal discharge was lessened, the nasal mucosa turned reddish and the swelling at the inferior turbinate abated or disappeared with improved or normal ventilation. Two weeks later, the nasal mucosa returned to normal in the majority of patients. A dull pain in the nasal cavity and maxillary tooth occurred among a few patients. These symptoms were relieved gradually after 1–2 weeks. The patients were followed up between 2 and 6 months and complications of nasal synechia, perforated nasal septum, atrophy of the nasal mucosa or dysosmia were not present. The excellence rate was 34.7% (25/72), the effective rate was 62.5% (45/72) and the ineffective rate was 2.8% (2/72). The total effective rate reached 97.2% (70/72).

## Discussion

AR is a chronic inflammatory disease occurring at the nasal mucosa. It is a type I allergic disorder mediated by IgE, in which various types of immunocytes and cytokines are involved. Rhinocnesmus, sneezing, excessive nasal discharge and nasal mucosal swelling are the main manifestations (12). Therefore, targets for the treatment of AR include submucosal over-reactive blood vessels, nerves, glands and locally-infiltrated immunocytes. With the development of basic research on the nasal cavity and paranasal sinuses, scholars have acknowledged that the nasal mucosal mucociliary system plays a critical role in maintaining the normal physiological functions of the nasal cavity. Damage to the system may result in complications, including a decrease in the immune function of the nasal mucosa, mycteroxerosis and backflow of nasal discharge. At present, the most commonly-used physiotherapies for AR in clinic include microwaves, lasers and radio frequency. These methods aim to reduce the nasal mucosal tissue according to the condition of the inferior turbinal mucosa, as well as the sources of the rhinal sensory and parasympathetic nerves (13–16). Although these therapies are able to achieve a certain short-term curative effect, they may additionally cause various degrees of damage to the epithelial layer of the nasal mucosa. An ideal therapy should lessen the functions played by the submucosal glands and immunocytes, and reduce the reactivity of the nasal mucosal blood vessels and nerves. An ideal therapy should also maintain the integrity of the anatomical structure and physiological functions of the nasal cavity, and be repeatable.

The mechanism underlying high-intensity focused ultrasound for the treatment of AR is that the power of ultrasound is focused on the nasal submucosal layer which contains numerous immunocytes, glands and blood vessels to cause punctate coagulative necrosis. Such necrosis leads to: i) decrease in the number of immunocytes which further reduces the numbers of cytokines and inflammation transmitters released by the immunocytes and induces the degranulation of mastocytes, which is equivalent to immunotherapy; ii) the direct destruction of the glands and decrease in gland secretion; iii) the destruction of mucosa deep-seated parasympathetic micro-gangliocytes and substance P nerve fibers, which reduces the excitability of cholinergic nerves as well as the release of vasoactive peptides, thereby exerting an anticholinergic effect; and iv) the occlusion of the blood vessels and formation of thrombi, which reduce exudation from the plasma and relieve the mucosal swelling. As focused ultrasound is able to maintain the integrity of nasal mucosal surface tissues, it will not damage the secretary function of the nasal mucosa or the normal function of cilia. Therefore, compared with other physiotherapies, high-intensity focused ultrasound has the advantages of higher safety, no incision, no foreign body implantation, no radioactivity or electromagnetic radiation and a lower complication rate. High-intensity focused ultrasound protects the rhinal structure and nasal mucosal function well without nasal synechia or backflow of nasal discharge. In addition, its operation is simple and may be performed repeatedly.

Two sources of inefficiency in the current study may be present. First, the range ultrasound is able to cover is limited due to the volume of the ultrasound tool bit and anatomical factors (including the narrow space of the nasal cavity and deflection of the nasal septum) for which the tool bit cannot contact a larger area of the mucous membrane. Second, an abnormal anatomical structure, such as the crest of the nasal septum or the skeletal protrusion of the inferior turbinate, denies the emitter window access to the treated area vertically and sufficiently during treatment, and a protruding spinous process often breaks the plastic ultrasound emitter window, resulting in water leaks from the tool bit. Thus, patients with abnormal anatomical structures, such as an excessively narrow space of the nasal cavity and severe deflection of the nasal septum, should be treated carefully. These abnormal structures may be used as the contraindications of ultrasound treatment.

Endoscopic high-intensity focused ultrasound may achieve an improved short-term curative effect for the treatment of AR. Compared with other methods, it possesses the advantages of fewer adverse effects, lower complication rate and higher safety. High-intensity focused ultrasound should be investigated as a novel effective treament method for AR in clinical practice.
